# Media coverage of climate activist groups in Germany

**DOI:** 10.1007/s10584-025-03959-8

**Published:** 2025-07-23

**Authors:** Fabian Dablander, Simon Wimmer, Jonas Haslbeck

**Affiliations:** 1 Institute for Biodiversity and Ecosystem Dynamics, University of Amsterdam, Amsterdam, The Netherlands; 2 Institute for Advanced Study, University of Amsterdam, Amsterdam, The Netherlands; 3 Independent Researcher, Munich, Fed. Rep. Germany; 4 Department of Clinical Psychological Science, Maastricht University, Maastricht, The Netherlands; 5 Department of Psychological Methods, University of Amsterdam, Amsterdam, The Netherlands

**Keywords:** Climate activism, Climate change, Polarization, Large language models, Fridays for future, Last Generation

## Abstract

Climate activist groups aim to address climate change by informing citizens about its risks and potential solutions, and by providing a way for citizens to engage in collective action to change policy. The effectiveness of climate activist groups, some of which engage in disruptive protests, is influenced by how they are portrayed by the news media. Using frequency analysis and GPT-4, we analysed all online news articles from major German newspapers in 2022 and 2023 about the two most prominent climate activist groups, Fridays for Future and Last Generation. A substantial proportion of the articles provides little information about the risks and solutions of climate change, especially when reporting on the more disruptive Last Generation compared to Fridays for Future, which primarily engages in legal protest. Last Generation is also portrayed more negatively, as more violent, and as more polarising. Right-leaning newspapers provide the least information about climate change and portray activist groups most negatively. We discuss the implications of our results for the media, activist groups, and future research.

## Introduction

Despite urgent warnings about the consequences of continuing on our current trajectory (Pörtner et al. 2023), global action on climate change remains inadequate (Boehm et al. 2023; UNEP 2024). There is a growing recognition that insufficient mitigation and adaptation to climate change is substantially due to resistance from actors who benefit from the status quo (e.g., Colgan et al. 2021; Geels 2014; Grubb et al. 2022; Stoddard et al. 2021). Overcoming this resistance and compelling governments and corporations to take decisive action against climate change requires bottom-up pressure from large parts of society (Fisher 2024; Temper et al. 2020; Thiri et al. 2022). A necessary ingredient for citizens to create such bottom-up pressure is that they are (a) aware of the severity of the risks and the adequacy of potential solutions and (b) willing to join groups that engage in collective action to change policy.

Climate activist groups aim to achieve these two goals through tactics ranging from legal marches to nonviolent civil disobedience. To make their actions and demands known to the general public, they heavily rely on the news media (Bevins 2023). How effective activist groups are with reaching the general public is strongly influenced by how the news media covers them, their actions, and the issue they advocate for (see Boydstun 2013; Huang et al. 2021; McCombs and Shaw 1972; Weaver et al. 2004; and Chinn et al. 2020 specifically for climate change). In addition, the solutions presented in the media shape the range of responses that people are willing to consider (Benford and Snow 2000). Through these mechanisms, the news media influences both public opinion and climate policy (Bromley-Trujillo and Poe 2020; Steurer et al. 2020). Trying to increase media reporting about the risks of climate change and the inadequacy of the current response has therefore become a central strategy of climate activist groups.

However, the extent to which climate activists are successful in informing the public through the news media is largely unclear. The success likely depends on the actions by the activists, but it likely also depends on the extent to which news reports about them actually cover the topic of climate change, and how the climate activist groups themselves are being portrayed, since the effectiveness of communication is influenced by the perceived legitimacy of the messenger (e.g., Pornpitakpan 2004; Wilson and Sherrell 1993). Relatedly, citizens are arguably less likely to join a group that is being portrayed negatively. Existing work focuses mostly on how climate change is generally covered in the news media (e.g., Bromley-Trujillo et al. 2023; Chinn et al. 2020; Trumbo 1996), on how activists frame climate change compared to the news media (e.g., Chen et al. 2023), or on a small aspect of the framing such as ageist delegitimisation of activists (Bergmann and Ossewaarde 2020). Other work focuses on the direct impact of climate activist groups on, for example, voting behaviour (Valentim 2023), environmental concern and climate change awareness (Brehm and Gruhl 2024; Kenward and Brick 2024; Ostarek et al. 2024), or policy change (Rogers et al. 2024). In terms of work focusing on the media, research indicates that politically right-leaning newspapers tend to amplify climate change sceptical voices more than politically left-leaning newspapers (e.g., Meier and Eskjær 2024; Painter and Ashe 2012), use more incendiary language in headlines (ISD 2020), and oppose climate action more strongly overall (Gabbatiss and Hayes 2024). Von Zabern and Tulloch (2021) stand out by analysing the frames selected German newspapers use when writing about the non-disruptive climate activist group Fridays for Future, concluding that their voice “is often reduced to apolitical testimonies”. Similarly, Scheuch et al. (2024) investigated the effect of different activist tactics and targets on media reporting, finding that actions that disrupted parts of the general public drew more press coverage and that right-leaning outlets covered climate actions more unfavourably. However, to our knowledge, no previous work has analysed (a) how climate change is being portrayed in news reports about climate activist groups, nor analysed (b) how the portrayal of climate activists differs between non-disruptive and disruptive groups.

Here, we provide the first large-scale analysis of news articles about climate activist groups, investigating both the portrayal of climate change and the groups themselves. Specifically, we analyse all relevant 4,216 online news articles published between 01/01/2022 and 31/12/2023 in seven major German newspapers — ranging from the cooperatively owned, politically left-leaning Taz.de to the privately owned, politically right-leaning Bild.de — about the two most prominent climate activist groups *Fridays for Future* (FFF) and *Last Generation* (“*Letzte Generation*” in German, LG). Reporting about these two groups far exceeds the reporting of any other climate groups in Germany. We focus on Fridays for Future and Last Generation because they represent contrasting approaches to climate activism. Fridays for Future, a global youth-led movement inspired by Greta Thunberg, organizes school strikes and large-scale national and global protests that (used to) draw millions to the streets, focusing on inclusivity and raising awareness. In contrast, Last Generation engages in nonviolent civil disobedience, primarily through disruptive road blockades, to pressure politicians into enacting stricter climate policies. While FFF’s broad appeal and moral authority make it widely accessible, its reliance on traditional protest forms may limit its capacity to disrupt entrenched systems. Conversely, LG’s confrontational tactics provoke stronger reactions but can polarise public opinion, potentially reducing its broader acceptance. Together, they span a spectrum of activism, making them suitable as case studies. We focus on Germany because it has one of the strongest climate movements in the world and an outsized influence on European climate policy. We end with a discussion of our results and implications for activist groups, the news media, and the general public.

## Methods

We begin by describing how we obtained the news articles using a scraping method we developed (Section 2.1) and how we analysed them using both traditional frequency analysis (Section 2.2) and the large language model GPT-4 (Section 2.3). We report on the validation of the latter in Section 2.4. To ensure data quality, we applied a filter described in Section 2.5, and we describe how we present and interpret our results in Section 2.6. For all details on our methodology, see Appendix A.

### Scraping online news articles

Our goal was to obtain all news articles in seven of the most important German online news websites in the years of 2022 and 2023 that were about climate activist groups or their actions. Specifically, we chose online newspapers Taz.de, Zeit.de, SZ.de, Spiegel.de, FAZ.net, Welt.de, and Bild.de because of their reach and because they cover political orientation from left to right. Bild.de and Welt.de are the newspapers with the largest reach in Germany with 12.24 and 3.62 million readers per day (combining print and online) in 2023, while SZ.de and FAZ.net take fourth and fifth place, with 2.46 and 2.12 million readers per day in 2023, respectively, according to Arbeitsgemeinschaft Media-Analyse, a well-known German organisation for media analysis.^1^ Spiegel.de is said to have the largest reach in print with 5.01 million readers.^2^ In terms of political orientation, Taz.de, Zeit.de, SZ.de, and Spiegel.de lean to the left, while FAZ.net, Welt.de, and Bild.de lean to the right (as assessed by https://ground.news/). There are differences within these two blocks, however, with Taz.de and Bild.de leaning more strongly to the left and right, respectively. Overall, the chosen set of newspapers provides breadth in political orientation. Our scraping approach captured all articles that included spelling variations of FFF and LG (for details, see Appendix A.2). To be able to investigate differences between the two climate activist groups, we excluded articles that mention both groups (7.8%).

### Frequency-based analyses

We applied two frequency-based analyses. In the first we analysed the frequency of all articles across time after subsetting the relevant articles (see Section 2.5). In the second analysis, we used the same subset and used word frequency analysis to calculate (a) how often ‘crisis’ or ‘emergency’ terms related to climate change were mentioned, (b) to what extent climate activist groups were negatively portrayed by referring to them with derogatory terms, and (c) to what extent the articles suggested that climate activist groups are polarising society. For more details, see Appendix A.3.

### GPT-based analyses

We used OpenAI’s GPT-4 Turbo (gpt-4-1106-preview) (OpenAI 2023), henceforth “GPT-4”, to answer 18 questions about each news article. We used zero-shot learning to infer qualitative properties of the reporting in newspaper articles by asking GPT-4 to read newspaper articles and to answer our questions for each of them. Our prompt used standard techniques to elicit higher-quality answers for our task (Chen et al. 2023). In particular, we used framing (“Imagine you are a political scientist studying...”) and steering (a specific JSON answer format is enforced). GPT-4 is prompted for each article individually, but with the full set of questions. The questions we asked were about (1) whether the article was a proper news article; (2) which type of article it was (interview, opinion piece, report,...); (3) the content of the article (about protest, legal issues,...); (4) whether the article is broadly about climate activism; (5) whether the article is mainly about climate activism; (6) whether the article is broadly about climate change; (7) whether the article is mainly about climate change; (8) whether climate change is portayed as a threat; (9) whether risks of climate change are discussed, (10) whether solutions to climate change are discussed; (11) whether the article mentions a disruption caused by a potential action of activist groups; (12) whether activists are portrayed as peaceful vs violent, coded on a 4-point Likert scale; (13) whether activists are portrayed as unhelpful or helpful; (14) whether activists are described as polarising society; (15) which actors are quoted in an article (activists, politicians, law enforcement,...); (16) whether the focus of the article is on the motivation of activists vs. the potential disruption caused by an action, scored on a 6-point Likert scale; (17) whether the article is focused on one activist group and if so which one; and (18) we asked for a 100 word summary of the news article.

In Appendix A.4 we describe GPT-4 in more detail and provide the exact wording of each prompt and descriptive statistics about both the answers GPT-4 gave and example articles for each of the available response options. It has been noted that training state-of-the-art LLMs requires large amounts of electricity (Faiz et al. 2024; Touvron et al. 2023). The energy cost and resulting carbon emissions from running GPT-4 *inference* to compute the results reported in this paper, however, are small: our best estimate is in the order of 10-100 kWhs used (see Appendix A.9). Discussions about whether the resources required to *train* LLMs are well spent are important, but naturally out of the scope of this paper.

### Validation of GPT-based analyses

We used GPT-4 to render the analysis of the large number of scraped news articles feasible. We validated the answers by GPT-4 by comparing it to the answers of three humans on 60 randomly sampled articles (see Appendix A.6). The validation showed high agreement between GPT-4 and human coders, underscoring the reliability of GPT-4 for this task. The use of large language models (LLMs) such as GPT-4 in content analysis has increased since these models have become much more powerful. For example, recent studies have demonstrated that they outperform humans in text-annotation tasks (Gilardi et al. 2023), and can exhibit performance similar to that of expert human raters for assessing the quality of texts (Chiang and Lee 2023) and even in data analysis (Cheng et al. 2023).

### Filtering data

To ensure that all our news articles are strictly about climate activist groups, additional filtering was necessary. This is because, while an article might include the term “letzte generation” (English: “last generation”), this may be in a context that has nothing to do with climate activism. In addition, since we intended to compare the activist groups FFF and LG, we needed to ensure that all articles were predominantly about one or the other activist group and not about both, climate activists in general, or other activist groups. To achieve the above we only included articles in the main analysis that were (a) proper news articles, (b) that were mainly about climate activism, and (c) that were predominantly either about FFF *or* LG.

For SZ.de we were able to identify the articles that were mirrored from the Deutsche Presse Agentur (DPA) news agency. We also noticed that SZ.de published a very large proportion of such mirrored articles compared to other newspapers. We therefore decided to only include original articles of SZ.de in the main analysis. For more details on this decision and the results of our analysis for the DPA articles mirrored by SZ.de, see Section B.6. For additional details on this filtering process, see Appendix A.5.

### Analyses and inference

We report the results of the frequency- and GPT-based analyses conditional on time, activist group, and newspapers. While our sample may miss a small number of articles due to one-off technical intricacies (see Appendix A.5), for all practical purposes we consider our sample to be equal to our target population of all articles on climate activists in 2022-23 in the seven considered online newspapers. Because we are directly observing the population, we do not need to use statistical inference, the only purpose of which would be to make inferences about that very population.

## Results

We identified 9, 056 online news articles in seven major German online newspapers that mentioned the climate activist groups *Fridays for Future* (FFF) or *Last Generation* (LG). In our main analysis, we only considered those articles that were proper news articles (e.g., no live tickers or podcast transcripts), that were mainly about climate activism, and that were predominantly either about FFF or LG (e.g., no articles about other groups or several groups) in order to avoid corrupting comparisons across activist groups (see Section 2.5). After this filtering we obtained our final sample of $N = 4,216$ news articles from Taz.de (435), Zeit.de (859), SZ.de (285), Spiegel.de (249), FAZ.net (162), Welt.de (786), and Bild.de (434). We also separately captured articles of the Deutsche Presse Agentur (DPA) from SZ.de (1, 006), which we analyse in Section B.6. Across newspapers, 84.3% of articles were reports, 3.4% interviews, 9.4% opinion pieces, and 2.8% other types of articles, and a large majority of articles (85.2%) were about LG. For more detailed descriptive statistics, see Section B.1.

### Number of published articles across time

**Fig. 1:**
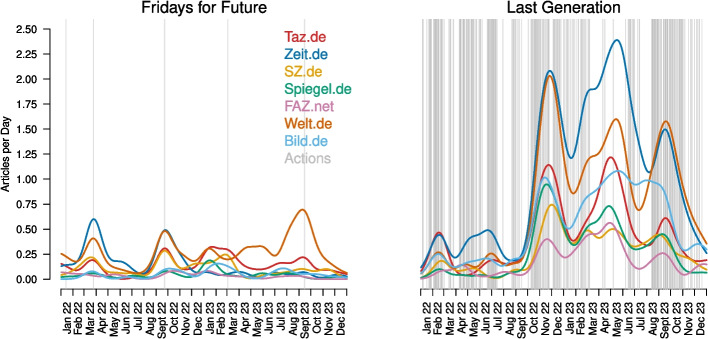
The number of articles published per day from January 2022 to December 2023. Results are shown separately for the seven newspapers (colours) and Fridays for Future (left) and Last Generation (right). Time series were smoothed with a Gaussian kernel ($\sigma =20$) to improve clarity. Actions of the activist groups are displayed as grey vertical lines

We first analysed how the number of articles about the two climate activist groups changed over time for the seven newspapers. Figure 1 shows the number of articles published per day about Fridays for Future (left) and Last Generation (right) separately for the seven newspapers in 2022 and 2023. For FFF, we see temporary increases in reporting in March 2022, September 2022, March 2023, and September 2023, which (except for the first one) correspond to the global climate strikes of Fridays for Future (grey vertical lines). However, the total number of articles does not permanently increase over time. Note that while FFF used to organize school strikes every Friday, this has diminished in recent years, with a shift in focus towards larger demonstrations and collaborations with other organizations, such as the labour union Verdi from 2023 onwards (Hasselbach 2024). Figure 1 does not include vertical grey lines for school strikes, because FFF does not send out press releases for those, making it difficult to know when and where they occurred. Irrespective, Fig. 1 shows that only FFF’s large protest drive media coverage. For LG, there is hardly any reporting in the beginning of 2022, since this is when the group started its first actions. We then see sharp increases in fall 2022, spring 2023, and again in fall 2023, coinciding with periods of many actions. These were extracted from the groups’ press releases (see Section A.8) and are shown as grey vertical lines. The low number of grey lines for LG in spring 2023 can be explained by two phenomena that led to fewer official press releases: police raids disrupted the group’s (online) operations and they had a higher focus on decentralised actions across Germany during this period.

Focusing on individual newspapers, we see that the volume of reporting of all newspapers is substantially driven by the actions of the activist groups. However, beyond this observation, we see large differences between newspapers. For example, Zeit.de provides the most reporting on climate activism overall, but its focus seems to have shifted away from FFF and towards LG at the end of 2022. Reporting of Welt.de, on the other hand, increased considerably for both climate activist groups.

### Portrayal of climate change

**Fig. 2:**
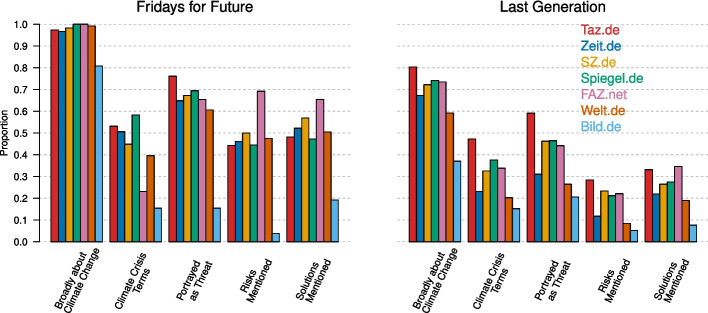
Portrayal of climate change in news articles about climate activist groups. Results are shown separately for the seven newspapers (colours) and Fridays for Future (left) and Last Generation (right). For the exact wording of all questions, see Section A.4

We first show how the topic of climate change is being portrayed in articles about the two climate activist groups. Using GPT-4 to answer questions about each news article, we assessed whether the articles were broadly about climate change, whether climate change was portrayed as a threat, and whether the risks of and solutions to climate change were mentioned (for details, see Section A.4). We also used frequency analysis to assess whether crisis terms such as “climate crisis” or “climate catastrophe” appeared in the text (see Section A.3). The proportions of times these questions were answered with “Yes” are shown in Fig. 2.

The first question captured whether an article was “broadly about climate change”, which is answered with “Yes” if it mentions climate change or topics related to it (for details, see Section A.4). We see that almost all articles (97.3%) about FFF were at least broadly about climate change. Among the articles about FFF, differences between newspapers were largely due to the politically right-leaning Bild.de, whose articles were less likely to be broadly about climate change (80.8%) than the other newspapers (average 98.2%). Considerably fewer articles about LG were broadly about climate change (63.6%). Differences between newspapers were more pronounced. For example, 80.4% of articles on the politically left-leaning Taz.de were broadly about climate change, while this was the case for 59.2% of articles on the politically right-leaning Welt.de and only 37.0% of articles on Bild.de. We see a similar pattern when looking at terms that emphasise the presence of an urgent crisis due to climate change, such as “climate crisis”, “climate emergency”, “climate catastrophe”, and “climate collapse” (for details, see Section A.3). These terms were used more often in articles about FFF (44.7%) than in articles about LG (26.5%). Differences between newspapers were larger than between activist groups: Taz.de mentioned one or more of the above terms in 48.7% of its articles, while Bild.de did so in only 14.2%. These differences are not trivially explained by differing lengths of articles in those newspapers (see Section B.2).

We next assessed the extent to which climate change was portrayed as a threat, whether its risks were mentioned, and whether potential solutions were mentioned. These questions were designed to be very lenient. For example, we already considered an article to portray climate change as a threat if it uses the term “climate crisis”; stating the mere fact that the earth’s temperature is rising was counted as a risk; and solutions were counted even if they were small given the scale of the problem (e.g., reducing food waste, introducing speed limits). For more details on the exact questions, see Section A.4. Overall, we found that climate change was being portrayed as a threat in 39.1% of the articles, risks were mentioned in 18.7% of articles, and solutions were discussed in about 25.9% of articles. Again, there were large differences across groups and newspapers. The percentage of articles portraying climate change as a threat and discussing risks and solutions was much higher for FFF (64.4%, 45.3%, and 49.9%) than for LG (34.8%, 14.1%, and 21.8%). The percentages were also considerably lower for the newspapers Spiegel.de, Welt.de and especially Bild.de, whose articles mentioned threats (49.8%, 31.8%, 20.3%), risks (24.5%, 14.6%, 5.1%), and solutions (30.4%, 23.8%, 8.3%) much less often than the other newspapers (average of all other newspapers 45.6%, 23.2%, and 30.7%, respectively).

Articles about LG were more often about protests than articles about FFF (see Section B.1), which raises the question whether differences between the groups are explained away by this fact. We investigated this by repeating all analyses only for those articles that report about a protest. In this analysis, the group differences are only somewhat attenuated (see Section B.7). We also assessed whether the differences between newspapers were larger than the differences between climate activist groups for all outcomes. We found that this was especially the case for “portrayed as a threat” and “solutions mentioned”, with much smaller differences for “broadly about climate change” and climate crisis terms. The differences for “risks mentioned” were larger between activist groups than between newspapers (for details, see Section B.3). Finally, we analysed the data separately for the years 2022 and 2023. We found that the results in both years are very similar to the combined results in Fig. 2. The largest difference was that most newspapers reported less on risks and solutions when reporting about FFF in 2023 (For details see Appendix B.8).

### Portrayal of climate activist groups

**Fig. 3:**
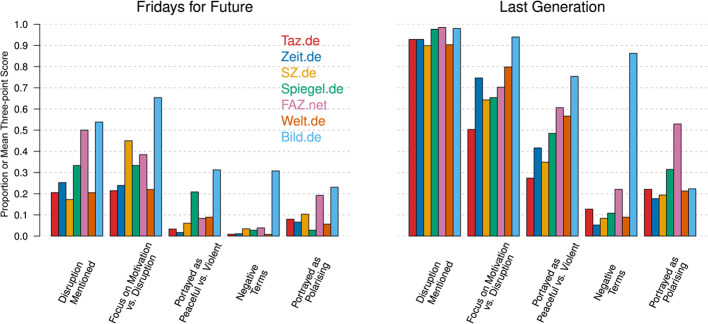
Portrayal of climate activists in the news media. Shows proportion of “Yes” answers to the questions. For the item “Portrayed as Peaceful vs. Violent”, peaceful is coded as 0 and violent as 1. The item “Focus on Motivation vs. Disruption” is three-point and coded as 0 = focus on motivation, 0.50 = equal focus, 1 = focus on disruption. For this item we display the mean across the three responses. Results are shown separately for the seven newspapers (colours) and Fridays for Future (left) and Last Generation (right). For the exact wording of all questions, see Section A.4

We next investigated how climate activist groups themselves were being portrayed. We used GPT-4 to assess whether the articles mentioned any disruption caused by the activists, focused more on the activists’ motivation or the disruption caused, portrayed the climate activists as peaceful or violent, or portrayed them as polarising society. These items were also answered with “Yes” or “No” with the exception of the question about motivation vs. disruption and the question about peaceful vs. violent. These questions were assessed with Likert scales which were trinarised and binarised to increase their validity (see Section A.4). We also used frequency analysis to assess whether articles used negative terms such as “climate terrorist” and “climate sadist” to describe climate activists (see Section A.3). In Fig. 3 we show the proportion of “Yes” answers for all items. For motivation vs. disruption we show the mean of the three-point variable (0 = focus on motivation, 0.50 = equal focus, 1 = focus on disruption). For the binary peaceful vs. violent question the coding is 0 for peaceful and 1 for violent.

The first two questions were about whether any disruption was mentioned and whether the focus of the reporting was more on the motivation or the disruption of the climate activist group. A focus on motivation would mean that the article in some way mentions the motivation of the activists, which could be the general topic of climate change or specific demands. In these questions, we see large differences between activist groups. In articles about FFF, disruption was mentioned on average in 25.5% of all articles. When analysing whether the focus was more on disruption or on the motivation of activists, we only considered those articles in which disruption was mentioned. This is because this contrast only makes sense if one could conceivably report on a disruption. For FFF, we found that 86.4% of articles were focused mostly on the motivation of activists, 9.5% were focused equally on disruption and motivation, and 4.2% were focused mostly on disruption. In articles about LG, disruption was mentioned in almost all articles (93.4%), and only in 13.7% of articles the focus was mostly on the motivation of the activist group, in 31.2% of articles equally on disruption and motivation, and in 55.2% of articles mostly on disruption. Bild.de was the newspaper which reported more disruption and focused much less on the motivation of the activists than other newspapers for both FFF and LG.

We also investigated to what extent activist groups were portrayed as peaceful or violent, whether they were described with derogatory terms such as “climate terrorist” and “climate criminal”, and whether the article explicitly portrayed the group as polarising society. Again, we found large differences across groups and newspapers. Despite the fact that FFF engages only in legal protests and advocacy, 9.1% of all articles portrayed them as violent. The more confrontational LG was portrayed as considerably more violent (49.8%). To what extent activist groups were portrayed as violent also strongly depends on the political leaning of newspapers, with 22.9% of articles of the left-leaning Taz.de describing activists as violent compared to 73.6% of articles of the right-leaning Bild.de.

Across all newspapers, 5.5% of articles used derogatory terms in articles about FFF, with 85.7% of these articles using “climate gluer” as the only negative term. As FFF does not use glue in their actions, this negative term is unlikely to refer to FFF activists themselves. The big outlier is Bild.de, which used negative terms in 30.8% of its articles about FFF (50.0% of which included “climate gluer” as the only negative term). Derogatory terms were used much more frequently for LG (on average 10.3% excluding Bild.de) and especially in Bild.de (86.3% of articles). Across all newspapers, on average 62.6% of all articles that included negative terms about LG used the term “climate gluer” as the only negative term.

In articles about FFF, less than 10% of articles across newspapers portrayed them as polarising society, with the exception of the politically right-leaning FAZ.net (23.1%) and Bild.de (19.2%). In articles about LG, about 20% of articles across newspapers portrayed them as polarising society, with FAZ.net and Spiegel.de being large outliers with 52.9% and 31.5% articles, respectively. We inspected a sample of FAZ.net articles, which showed that it is indeed publishing a lot of opinion pieces that are critical of FFF and especially LG.

We also investigated which actors were quoted in news articles. In articles about FFF, the top three most quoted actors were activists (84.4% of articles), politicians (28.3%), and business representatives (16.6%). In articles about LG, the top three most quoted actors were activists (43.6%), law enforcement (41.8%), and politicians (39.0%). For details, see Section B.5.

We found that the differences between newspapers were larger than the differences between climate activist groups for negative terms, and the questions of motivation vs. disruption and being portrayed as peaceful vs. violent. The opposite was true for whether disruption was mentioned and whether activists were portrayed as polarising (for details, see Section B.3). We also analysed the data separately for the years 2022 and 2023 and found that the results in both years are very similar to the combined results in Fig. 3. The largest difference was that most newspapers focused more on disruption when reporting about FFF in 2023 (for details see Appendix B.8). Finally, we also calculated the similarities between all of our outcome measures, which we discuss in Section B.4.

## Discussion

This study investigated how the German news media covers the two most prominent climate activist groups in Germany, Fridays for Future and Last Generation. We found that a considerable proportion of articles did not contextualise the groups’ actions within the wider discourse on climate change, instead tending to focus on the protesters’ disruption or legitimacy. More than two-thirds of news articles did not mention climate crisis terms in their coverage, and less than a quarter mentioned risks or solutions. This is in line with the “protest paradigm”, which suggests that media representations often delegitimise or marginalise protest groups, focusing on the spectacle of the protest (such as violence, disruption, or dramatic confrontations) rather than the underlying social or political issues they aim to address (e.g., Caren et al. 2020; Di Cicco 2010; McLeod and Hertog 1999); see Cooper (2023) for a similar finding regarding US television coverage of climate protests. This is also reflected in differences between FFF and the more disruptive LG. Compared to articles about LG, articles about FFF tended to focus more on the motivation of the protesters than the disruption caused. LG, which received significantly more overall coverage than FFF, was also more frequently portrayed as violent and as polarising society. These differences in reporting are consistent with the “activist’s dilemma”, which describes the phenomenon that less disruptive actions tend to receive less media coverage (Amenta et al. 2009; Macdonald 2023; Scheuch et al. 2024), while more disruptive actions tend to be reported on less favourably for the activist group (Feinberg et al. 2020; Gitlin 2003; Scheuch et al. 2024).

While the reporting differed across the two activist groups, the largest differences in reporting were observed across newspapers. There was a large contrast in reporting between Taz.de, Zeit.de, SZ.de, Spiegel.de, and FAZ.net on the one hand, and Welt.de and especially Bild.de on the other hand, the latter of which lean more to the right politically (see Section A.2). On all accounts, Bild.de provided the least context about climate change and portrayed both activist groups as more violent, focusing more on the disruption than motivation, and more frequently using negative terms to refer to them. Note that this observation is not simply explained by shorter article lengths in Bild.de, which are actually similar to those in Zeit.de (see Section B.2). This finding is consistent with previous research showing that conservative news media reports more negatively on Extinction Rebellion in the UK (Scheuch et al. 2024), uses more incendiary language in headlines (ISD 2020), and tends to oppose climate action more strongly overall (Gabbatiss and Hayes 2024). There is extensive research on how fossil fuel interests have pushed reporting skeptical of climate change in the past (e.g., Farrell 2016), and their strategy now also includes vilifying climate activist groups. For example, the Atlas network, a collection of neoliberal think tanks (Djelic and Mousavi 2020), has been pushing a narrative that portrays climate activists as terrorists, influencing politicians and media outlets (including Welt.de and Spiegel.de) in Germany and elsewhere to use this framing (Almiron et al. 2020, 2023; Westervelt and Dembicki 2023).

Our results have implications for news media, citizens, and activist groups. If one accepts the scientific consensus that the climate crisis needs to be urgently addressed as well as the significant scale of the transformations required (Pörtner et al. 2023; UNEP 2023), one may agree that the news media should better inform citizens about climate change, its risks and solutions, and the necessary systemic transformations. While it is difficult to reach a consensus on how much information about climate change should be included in a news article about climate activists, such articles certainly would provide one opportunity to better inform citizens. Our results may stimulate discussion among journalists and editors about how to adequately report on climate change and climate activist groups. To gauge whether the observations in this study are exclusive to reporting on climate activist groups, a fruitful avenue for future work would be to compare other climate-related reporting across newspapers (e.g., on United Nations Climate Change Conferences). Our results also raise the question which factors influence the editorial decisions of newspapers. For example, we found that Taz.de, which is a cooperative and entirely funded by its readers, is the newspaper that provides the most context about climate change when reporting on climate activist groups. In contrast, Bild.de is controlled by a few parties, some of which have expressed climate skeptic views (Gilbert and Stark 2023). Our results may contribute to a discussion about how to effectively reduce the outsized influence of such individuals on the public debate on climate change and climate activism. We also believe that our work and similar future work are an important way to inform citizens about the leanings of different outlets and might thereby help them stay informed but not be biased towards any one viewpoint.

Our results also have implications for the strategy of climate activist groups. Central goals of FFF and LG are to (a) convince the public of the need for stronger climate policy and to (b) mobilise citizens to join them in collective action to push for such policy. Since we found that coverage of climate activist groups frequently does not convey a sense of urgency to act on climate change, their success in achieving (a) is likely limited based on these reports alone. However, it may well be that while reporting on actions does not include adequate coverage of the risks and solutions to climate change, they spark a wider public discussion and lead to more coverage of climate change overall (c.f., Dunivin et al. 2022). We consider these important questions for future research. Regarding (b), because news coverage of the climate activist group tends to be negative, it may well be that fewer people join the group than if coverage would be positive. In addition to the nature of disruptive protests, the negative media coverage is likely a key reason why only about a third of the German public say that they support the climate movement (More in Common 2023). Activists, of course, also have an influence on the type of coverage too, and it may well be that actions that primarily disrupt the public are fundamentally unable to elicit mass support (Badullovich et al. 2024), which may suggest strategic rethinking (e.g., Huber 2022; Young 2024; Young and Thomas-Walters 2024). However, the “activist’s dilemma” suggests that non-disruptive protest, while possibly generating more favourable coverage, will lead to less coverage and exposure overall, and thus potentially fewer people becoming new active members of the group. In line with this notion and our findings, a recent representative poll suggests that 48% (24%) of the German public have a very negative (somewhat negative) opinion about LG (Davis et al. 2023). It is unclear which of these factors (positive coverage or overall coverage) dominates in increasing the size of the climate activist group, and hope that future research can address this question (Fisher et al. 2023; Ozden and Glover 2022).

We have presented a novel and validated methodology for the large-scale analysis of media reporting which only scratches the surface of the rich information contained in news articles. Authors of articles, whether consciously or not, influence the reader’s opinion on a given subject in many subtle ways, including word choice, presenting only one perspective and not another, selectively presenting data or quoting individuals, or choosing a positive or negative framing (for a concrete example, see Section A.7). We believe that future iterations of our methodology, perhaps based on more powerful AI technology, will be able to capture these more subtle aspects of framing at scale.

The conclusions that can be drawn from this study are subject to a number of limitations. First, while we focused on seven of the largest German online newspapers, there are of course many more smaller and regional outlets whose reporting may differ from the large newspapers we considered. However, we chose to focus on big news outlets because of their agenda setting function — they are more likely to shape public opinion and public debate than smaller outlets (McCombs and Shaw 1972). Moreover, we did not consider radio and television (Cooper 2023; Tschötschel et al. 2022) or social media, which has become a highly relevant tool for social movements (e.g., Mundt et al. 2018) and a significant source of news for many (Newman et al. 2017). Further research in these domains that would complement our results would be of great interest. Finally, our study was limited to German climate activist groups and German news media. However, we believe that the differences in reporting on confrontational versus less confrontational activist groups by politically left-leaning versus politically right-leaning newspapers will at least partially generalise to countries that are similar to Germany (see e.g. Scheuch et al. 2024). Many Western countries are home to both confrontational and less confrontational climate activist groups, for instance. We thus encourage others to apply our methodology to additional countries and assess to what extent our findings generalise.

### Conclusion

To what extent social movements can bring about change is strongly mediated by the news media. In the first large-scale study of media coverage of climate activist groups in Germany, we found that reporting often did not discuss the risks and solutions to climate change and that the more confrontational Last Generation received more coverage overall but was portrayed less favourably, especially by newspapers leaning towards the political right. We hope that our results and methodology can help increase our understanding of the complex landscape in which journalists report on climate change and climate activist groups seek to effect change.

## Figures and Tables

**Fig. 4:**
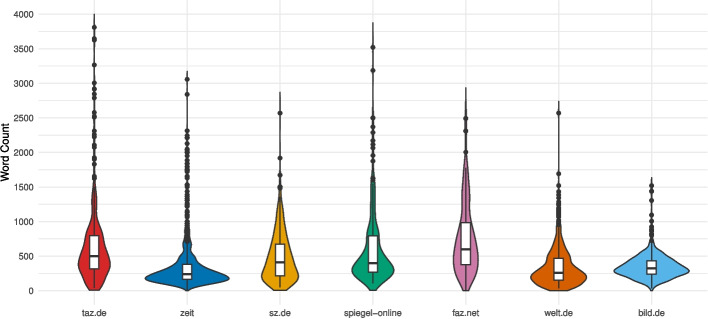
Violin plots of article length (in number of words on x-axis) for the seven newspapers considered in the main text. The violine plots show a density estimate of the empirical distribution. The boxes indicate the 25%, 50% (Median), and 75% quantiles

**Fig. 5:**
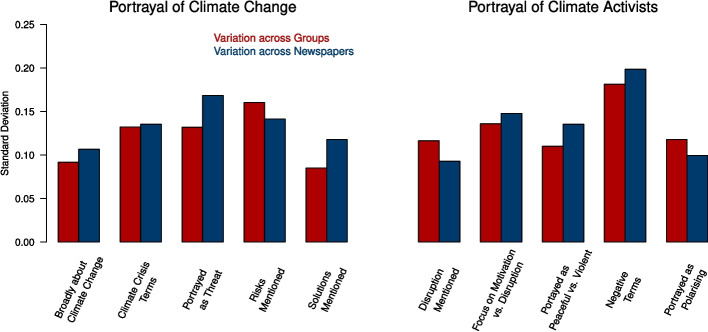
Standard deviation across groups (red) and across newspapers (blue) for portrayal of climate change (left) and portrayal of climate activists (right)

**Fig. 6:**
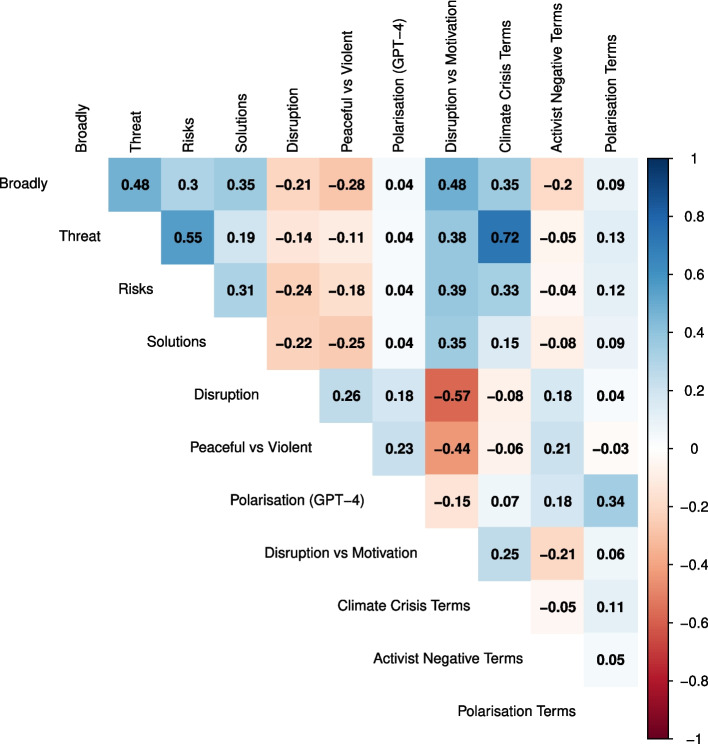
Correlation (Kendall’s $\tau$) of all outcomes across newspapers and activist groups

**Fig. 7:**
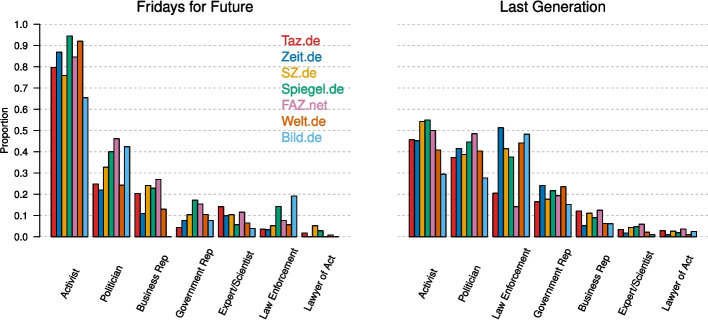
Proportion of articles in which certain actors are mentioned in the article. Results are shown separately for the six newspapers (colours), and the activist groups Fridays for Future (left) and Last Generation (right). Missing bars indicate a proportion of zero

**Fig. 8:**
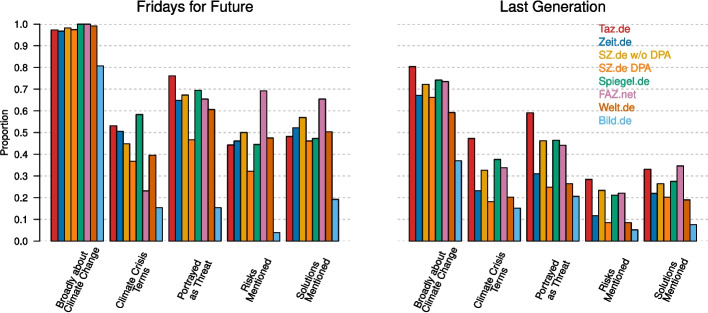
Portrayal of climate change in news articles about climate activist groups. The proportion of “Yes” answers to five questions capturing in which way the topic of climate change is reported on in the article. Results are shown separately for the seven newspapers (colours), and the activist groups Fridays for Future (left) and Last Generation (right). This is the same figure as Fig. 2 in the main text, but with articles of SZ.de split in articles written by their own journalists (SZ.de no-DPA) and articles copied from DPA (SZ.de-DPA)

**Fig. 9:**
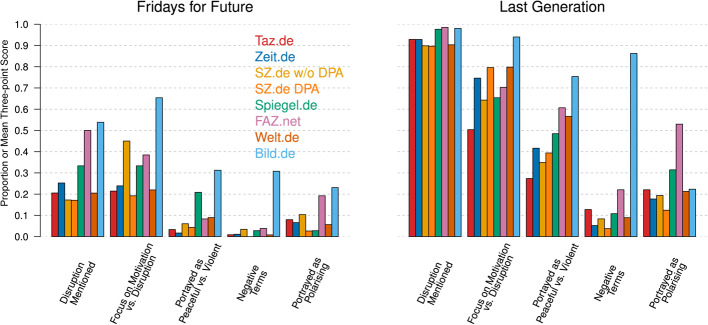
Portrayal of activists in the news media. The y-axis shows proportion of “Yes” answers to questions. The exception are the items “Focus on Disruption vs. Motivation” and “Portrayed as Peaceful vs. Violent” which were Likert scales which we normalised to [0, 1]. Results are shown separately for the seven newspapers (colours), and the activist group Fridays for Future (left) and Last Generation (right). This is the same figure as Fig. 3 in the main text, but with articles of SZ.de split in articles written by their own journalists (SZ.de no-DPA) and articles copied from DPA (SZ.de-DPA)

**Fig. 10:**
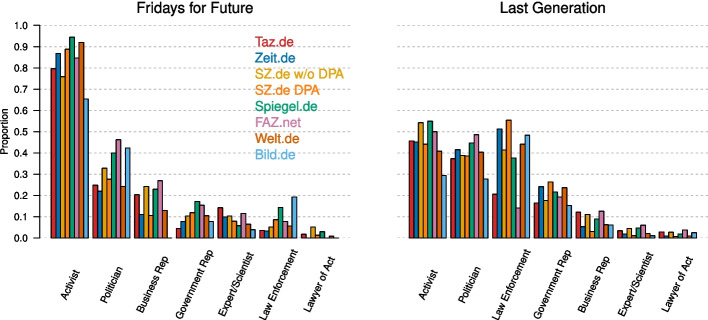
Proportion of articles in which certain actors are mentioned in the article. Results are shown separately for the seven newspapers (colours), and the activist groups Fridays for Future (left) and Last Generation (right). Missing bars indicate a count of zero. This is the same figure as Fig. 7 from Section B.5, but with articles of SZ.de split in articles written by their own journalists (SZ.de no-DPA) and articles copied from DPA (SZ.de-DPA)

**Fig. 11:**
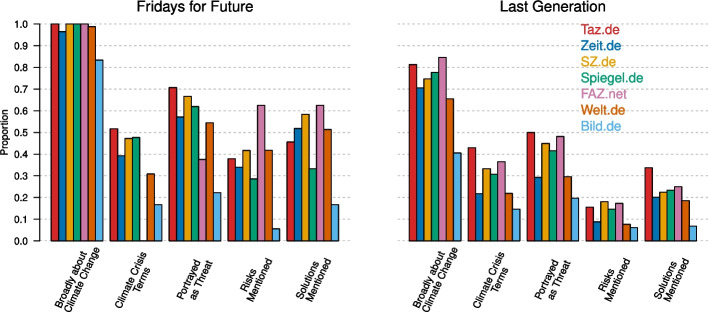
Same as Fig. 2 but computed only on those articles that are about a specific protest

**Fig. 12:**
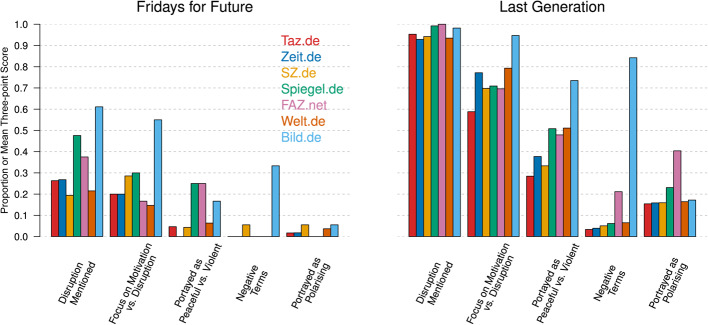
Same as Fig. 3 but computed only on those articles that are about a specific protest

**Fig. 13:**
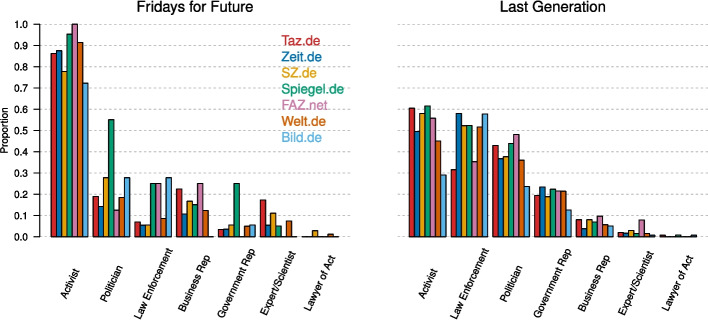
Same as Fig. 7 but computed only on those articles that are about a specific protest

**Fig. 14:**
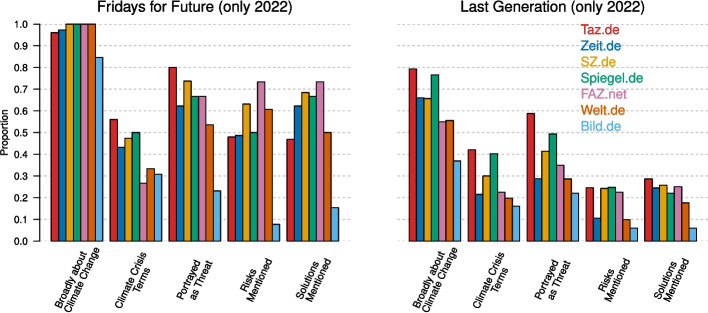
Same as Fig. 2 but computed only on the articles from 2022

**Fig. 15:**
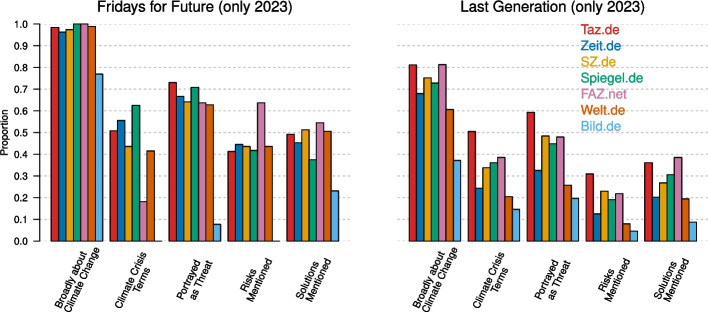
Same as Fig. 2 but computed only on the articles from 2023

**Fig. 16:**
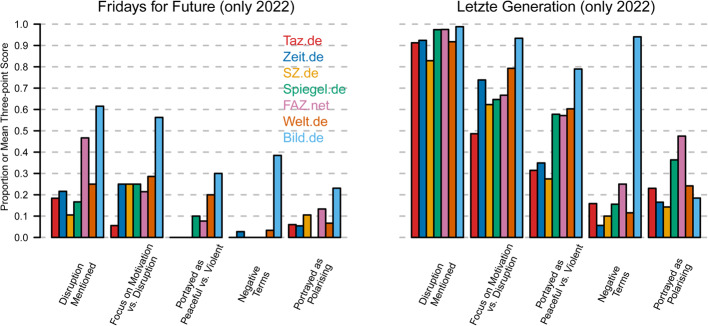
Same as Fig. 3 but computed only on the articles from 2022

**Fig. 17:**
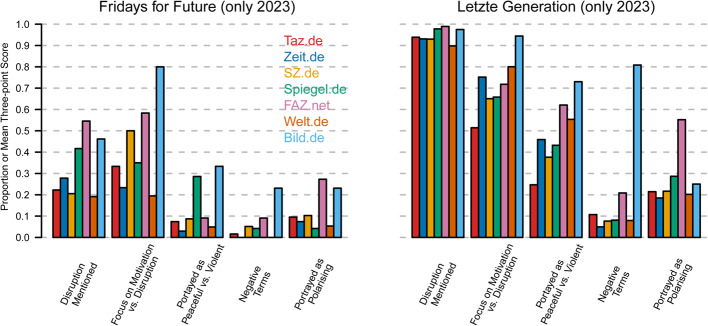
Same as Fig. 3 but computed only on the articles from 2023

## Data Availability

All data used, except the full article texts, are available at https://github.com/jmbh/ClimateActivistCoverage.

## A Extended method

This section describes the methodology we used to obtain and analyse news articles. We first describe in Section A.1 how the existing data bases for news articles, MediaCloud and LexisNexis, are incomplete, which motivated our own approach to obtaining news articles. Our own methodology consisted of five steps: First, relevant newspaper articles were identified and downloaded from the web (Section A.2). Second, simple frequency-based analyses were applied (Section A.3). Third, we used a modern machine learning tool to answer more complex semantic questions about the article texts (Section A.4). Fourth, based on static criteria and answers to the semantic questions, we filtered articles for relevance (Section A.5). Fifth, we evaluated the validity of the previous steps by manual analysis (Section A.6).

### A.1 MediaCloud and LexisNexis provide incomplete data

To find relevant articles, we initially wanted to use MediaCloud (https://www.mediacloud.org/), which allows searching global news media and which has previously been used for the analysis of climate activist groups (e.g., Chen et al. 2023). For Germany, MediaCloud has collections of 68 national news media and 258 state and local news media. However, when searching for articles that include “Last Generation” in their collection of national news media, we retrieve a total of just 478 articles. For SZ.de and Bild.de, we find a total of just 4 and 14 articles, respectively. That is by multiple orders of magnitude fewer than what we found with our custom scraping tool. The main reason for this is that MediaCloud uses RSS feeds to store the articles in real time, but their tool for doing this has suffered problems in the past (MediaCloud, personal communication). This cannot be fixed with their methodology retrospectively, because RSS feeds are short-lived. In addition to having very few articles about climate activist groups, it is also unlikely that the articles that they have are a representative sample of the total population of articles. This means that data on German climate activist groups from MediaCloud are unreliable and likely biased, and their analysis would likely lead to incorrect conclusions. We therefore consider using MediaCloud for the analysis of reporting on climate activist groups in Germany — and potentially for other topics as well — as problematic.

We also assessed whether LexisNexis (https://www.lexisnexis.com), which is part of the RELX corporation (formerly Reed Elsevier) and sells data analytics products to which universities often have access, would provide good enough access to articles published in major German newspapers. LexisNexis has access to many German newspapers, but their coverage seems at times incomplete, too. For example, they do not seem to have access to FAZ.net articles at all, and, for example, for Zeit.de we only found 470 articles for LG and FFF combined compared to our 1,659 in total (859 after applying filters) and for Taz.de only 795 compared to our 1,030 in total (470 after applying filters). To get the full picture for a particular newspaper, we thus consider our approach of scraping news articles directly the gold standard.

### A.2 Scraping online news articles

We scraped online news articles from seven major newspapers in Germany: Taz.de, Zeit.de, SZ.de, Spiegel.de, FAZ.net, Welt.de, and Bild.de. For FAZ.net, Spiegel.de, Zeit.de, and SZ.de, we used the search function on their respective website to identify relevant articles. For Bild.de, Taz.de, and Welt.de, we used Google News to identify relevant articles. We uniformly asked for exact matches to the action groups’ names. We account for the fact that Last Generation (“Letzte Generation”) and Fridays for Future sometimes appear in spelling variations (e.g. “Aufstand der letzten Generation” or “Fridays-for-Future”).

### A.3 Frequency-based analyses

We applied two frequency-based analyses. In the first analysis, we analysed the frequency of all articles across time after subsetting the relevant articles (see Section A.5.) In the second analysis, we used the same subset and used word frequency analysis to calculate (a) how often ‘crisis’ or ‘emergency’ terms related to climate change were mentioned, (b) to what extent climate activist groups were negatively portrayed, and (c) to what extent the articles suggested that climate activist groups are polarising society. To answer (a), we counted how often the terms ‘Klimakrise’, ‘Klimakatastrophe’, ‘Klimanotstand’, and ‘Klimakollaps’ appeared in the main body of the article. To answer (b), we searched for the terms ‘Klimakleber’, ‘Klimachaot’, ‘Klimaterrorist’, ‘Klimakriminell’, ‘Klimaradikal’, ‘Klimasadist’, ‘Klimasektierer’, ‘Klimakröte’, and ‘radikaler/radikale Klimaaktivist’. We took into account that the terms might be spelled as e.g. ‘Klima-krise’ or ‘Klima-kleber’. We used case-insensitive matching throughout. To answer (c), we used the following regular expression:

### A.4 GPT-based analysis

We found that GPT-4 is able to follow this format almost always (99.5%). What is more, by inspecting GPT-4’s answers, we observed that it is very robustly ignoring typical artefacts in the input texts. These include teaser blocks for other articles, advertisements, and data privacy disclaimers.

In what follows, we provide all questions used in our study in addition to a short description of how GPT-4 answered the questions. For the filtering questions (whether the text is a proper news article (A.4.1), whether it is mainly about climate activism (A.4.5), what action group its focus is (A.4.16)) and the question asking whether the text is broadly about climate activism (which is highly dependent on whether it is mainly about climate activism (A.4.4)), we provide statistics using the set of articles on which all filters have been applied except the filter that is under consideration. For all other questions, we provide statistics and examples using the completely filtered data set ($N = 4,216$). All example articles used in the question descriptions were sampled using a single seed to avoid cherry-picking. In Section A.6 we show that the responses of GPT-4 and human raters are very similar, validating our approach.

#### A.4.1 Proper news article

The exact wording of the prompt was:

We only used articles in the final analysis for which the answer to this question was yes, which occurred in 98.4% of cases. Out of the articles that were classified as not being a proper newspaper article many were podcasts,^3^ videos,^4^ or about TV programs.^5^

#### A.4.2 Type of article

The exact wording of the prompt was:

We collapsed the type “analysis” ($n = 52$) into “report”. Most articles were classified as reports (87.2%), followed by opinion pieces (7.4%), interviews (3.1%), and other (2.3%). Randomly selected examples for “other”articles include reporting on criticism of LG by a left-leaning politician,^6^ a report about a conversation between a Friday for Future activist and a business representative,^7^ and a table of contents page.^8^

#### A.4.3 Content of article

The exact wording of the prompt was:

Most articles were classified as reporting about a protest (65.6%), followed by “other” (19.7%) and then reporting about legal issues (14.7%). The articles that are classified as reporting about a protest^9^,^10^,^11^ or about legal issues^12^,^13^,^14^ are often straightforward. The “other” articles could be about a variety of topics, for example about FFF activists “manipulating” their grandparents to vote for the Greens,^15^ successful negotations of LG with the mayor of Hannover,^16^ and LG’s demand of a citizens’ assembly.^17^

#### A.4.4 Broadly about climate activism

The exact wording of the prompt was:

The large majority (93.6%) of articles were broadly about climate activism. We contrast this question with the next one. Note that for this and the following question, we use the data set on which all filters where applied except “mainly about climate activism”.

#### A.4.5 Mainly about climate activism

The exact wording of the prompt was:

More than half of all articles (61%) were classified as being mainly about climate activism. There were no articles that were mainly about climate activism but not broadly about it. Out of the 5,641 articles that were broadly about climate activism, 3,675 (65.1%) were also mainly about it. Examples of articles that were mainly about climate activism include a report about the spraying of a Christmas tree by LG,^18^ the spraying of the office building of the German chancellor,^19^ and roadblocks in Hamburg.^20^ Articles that were categorised as not being mainly about climate activism include an interview with a politician about asylum seekers,^21^ a speech by the German president,^22^ and a report about the party conference of the Greens in Berlin.^23^

#### A.4.6 Broadly about climate change

The exact wording of the prompt was:

A considerable amount (71.5%) of the filtered articles were classified as being broadly about climate change or the climate crisis. Examples of such articles include reporting on LG activists who were sentenced to jail and which states their motivation to fight against the “climate catastrophe”^24^ and an interview with an FFF spokesperson who said that the government lied about climate in the run-up to the elections.^25^ Articles that are not classified as being broadly about climate change include reporting on a politician who wants to put LG activists into preventive detention^26^ and reporting about protests that does not refer to climate change at all.^27^

#### A.4.7 Mainly about climate change

The exact wording of the prompt was:

Only 15% of articles were classified as being mainly about climate change. There were no articles that were mainly about climate change, but not broadly about it. Examples of articles that were mainly about climate change include one that is critical of degrowth,^28^ one about regular climate meetings inspired by FFF,^29^ and an analysis about food waste, a law towards furthering the prevention of which was one early demand of LG.^30^ Articles that were not mainly but broadly about climate change include reporting on the refusal of Greenpeace, LG, and FFF to have a stand at an auto show^31^ and an interview with a psychologist about the structure and strategy of LG.^32^

#### A.4.8 Climate change as a threat

The exact wording of the prompt was:

38.5% of articles were classified as portraying climate change / the climate crisis as a threat. This occurs when terms such as “climate catastrophe” or “climate collapse” are used when quoting activists and describing their motivation^33^,^34^,^35^ or if somebody else is quoted using those terms.^36^ However, 30% of articles that are classified as portraying climate change as a threat do not include any crisis- or emergency-related terms. One such article is about a politician criticizing both FFF and LG, but noting that if we do not keep to 1.5$^{\circ }$C catastrophic consequences will ensue,^37^ and another one reflects on five years of FFF, who are quoted as saying that profits are still put above human lives.^38^ Example articles that are not classified as portraying climate change as a threat are about violence against LG protesters,^39^ the city of Munich banning protests of LG,^40^ and the German attorney general saying LG is not a terrorist organization.^41^

#### A.4.9 Risks of climate change

The exact wording of the prompt was:

18.5% of articles were classified as mentioning the risks of climate change. Examples of such articles include one about protesters gluing onto a painting, quoting the activists as saying that there will not be a secure future under a two or four degree hotter Earth^42^ and one about LG’s spraying of the monument of the German constitution, quoting the activists that in the coming “climate hell” human dignity, human freedom, and fundamental rights will seize to exist.^43^ Examples that are not classified as mentioning risks include reporting about a protest where indeed no risks were mentioned^44^ and reporting about a protest that does mention that the “climate catastrophe” is the greatest threat to humans, society, and democracy but mentions no concrete risks.^45^ Overall, the correlation between the threat and risk question is Kendall’s $\tau = 0.55$ (see also Section B.4).

#### A.4.10 Solutions for climate change

The exact wording of the prompt was:

In the filtered data (see Section A.5), GPT-4 responded with “not applicable” in 0.4% of articles, in 72.3% no solutions were mentioned, and in 27.2% solutions were mentioned. Articles in which GPT-4 responds that no solutions are mentioned indeed include no reference to any kind of solution to climate change. Examples are articles in Taz.de,^46^ Zeit.de,^47^ Spiegel.de,^48^ and Bild.de.^49^

Articles that mention solutions include an article of Taz.de that is about how factory workers demand more climate friendly manufacturing.^50^ In an article of Zeit.de, the author repeats the demands of Last Generation for a 9-euro ticket for local public transport and a speed limit on highways.^51^ An article on SZ.de quotes an activist saying that there are many ways in which Germany can become independent of Russian oil.^52^ In an article on Spiegel.de, the demands of Last Generation for a 9-euro ticket for local public transport, a speed limit on highways, and the creation of citizen assemblies is repeated.^53^ In an article on Welt.de, Luisa Neubauer from FFF criticizes the climate politics of the government and says that especially the transportation ministry is lagging behind and mentions some examples. An article on Bild.de covers the activist Katja Diehl who discusses how we have to reconsider our ideas around the usage of cars.^54^ Interestingly, hardly any articles discuss the system level changes that are likely required to address climate change.

#### A.4.11 Disruption caused by activists

The exact wording of the prompt was:

79.3% of articles are classified as mentioning any disruption caused by the activists. Examples include an interview of a LG spokesperson about a big upcoming action^55^ and a report about an action where activists glued to a street to block traffic to an airport.^56^ Examples that are classified as not mentioning any disruption include an article about an FFF strategy meeting^57^ and a global climate strike by FFF that brought more than 200,000 people on the streets in Germany but did not mention any disruption.^58^

#### A.4.12 Portrayal of activists as peaceful vs. violent

The exact wording of the prompt was:

Most articles described climate activist groups as “mostly peaceful” (46%) followed by “mostly violent” (38.1%), “peaceful” (10.8%), and “violent” (5%). Only in 0.1% of cases did GPT indicate that this cannot be decided based on the article.

Typical articles labelled as “peaceful” report on legal protests, but do not explicitly describe activists as peaceful. One article by Bild.de with a “peaceful” verdict describes a symbolic FFF protest in Hannover using toboggans.^59^ Articles described as “mostly peaceful” fall into a broader range, but typically discuss or mention disruptive action forms such as roadblocks, but do not explicitly refer to these as violent or criminal acts. An early in-depth article about LG by SZ.de serves as an example.^60^ An article by FAZ.net^61^ criticises LG for being too radical, and mentions protest forms of sabotage, but without describing these explicitly as a form of violence.

#### A.4.13 Activists unhelpful vs. helpful

The exact wording of the prompt was:

In the filtered data (see Section A.5), GPT-4 responded with “not applicable” in 84.6% of articles. This is not surprising, since we explicitly require that either the author of the article or someone being quoted makes a direct statement about whether the activist group is helpful or unhelpful. Of the remaining 15.4%, GPT-4 indicated “unhelpful” in 12.5% of articles, and “helpful” in 2.9% of articles.

Examples for articles in which climate activists are portrayed as “unhelpful” include a DPA report published in Taz.de where German president Frank Walter Steinmeier directly criticises LG by saying that activist groups should engage in climate activism without antagonizing others.^62^ An article in Zeit.de does not explicitly say that LG is unhelpful for addressing climate change, but it contains very strong negative statements by the police union about the activist group.^63^ In an article in SZ.de, German chancelor Olaf Scholz directly criticises LG by saying that their form of protest is inappropriate and dangerous.^64^ In a Spiegel.de article, former Bild.de journalist Nikolaus Blome writes a scathing critique of FFF, which strongly implies that their actions are unhelpful for addressing climate change^65^ (for a more detailed analysis of this article, see Section A.7). In an article in FAZ.net, a journalist directly says that LG are “doing it wrong” and that their tactics are inappropriate.^66^ In an article on Welt.de, their editor-in-chief Ulf Poschardt calls LG activists extremists and celebrates that the police is taking stronger measures against them.^67^ Finally, Bild.de reports about a porn star who got in a traffic jam caused by LG and is venting about them.^68^

Examples for articles in which climate activists are portrayed as “helpful” include an article on Taz.de, which discusses FFF with quotes from social movement researchers and clearly implies that the activist group had a positive impact, for example on creating awareness about climate change in the general public.^69^ A Zeit.de article discusses the response of UN general secretary António Guterres to the house searches of the police of LG members, saying that climate activist are dearly needed.^70^ An article on SZ.de discusses the resilience of FFF in the face of the corona pandemic and quotes a climate researcher saying that FFF was important for raising public awareness about climate change.^71^ In an article on Spiegel.de, the author makes the argument that LG is helpful in that they force the general public to come to terms with climate change.^72^ In a FAZ.net article, a journalist interviews Sebastian Vettel and FFF leader Luisa Neubauer, the latter of which predictably makes the case that FFF is helpful for addressing climate change.^73^ An article in Welt.de reports on an action of FFF and quotes a local politician who says how important the action is for raising awareness about climate change.^74^ In an article in Bild.de, the author quotes António Guterres supporting climate activists.^75^

Since a large proportion of articles were classified as “not applicable”, we decided not to report this variable in the main text.

#### A.4.14 Activists and polarization

The exact wording of the prompt was:

In the filtered data (see Section A.5), 79.7% of articles were classified as articles in which climate activist groups were *not* described as polarising, and 20.3% of articles were classified as doing so.

Articles that were not classified as portraying climate activist as polarising include an article in Taz.de reports on the Russian climate activist Ashak Makichyan.^76^ An article in SZ.de reports on road blocks of LG in Munich.^77^ And Bild.de reports on legal issues related to LG.^78^

Articles that were classified as portraying activist groups as polarising include an article on Taz.de comments on how the political centre and right portray climate activists as terrorists, radicals and extremists.^79^ In a Zeit.de article, a journalist discusses why climate activists do not find acceptance in certain parts of the general public.^80^ An article in SZ.de explicitly states that the actions of LG are polarising society.^81^ In an article in Spiegel.de, the author discusses how the president of the Federal Office for the Protection of the Constitution says that LG is not an extremist group, and the article goes on how many in the general public to not support the actions of LG.^82^ An article in FAZ.net summarises discussions around the legitimacy of climate activist groups in Germany that took place in a German talk show.^83^ In an article in Welt.de, a FFF leader is quoted who says that the current debate about climate change is polarising society.^84^ Finally, an article in Bild.de describes climate activists of the group LG as antidemocratic and extremist.^85^

#### A.4.15 Quoted actors

The exact wording of the prompt was:

In the filtered data (see Section A.5) we obtained the following frequencies for the different groups being classified as quoted: activists 49.9% of articles, lawyers of activists 1.5%, law enforcement 39.3%, politicians 37.3%, government representatives 20.3%, business representatives 7.7%, and experts or scientists 3.2%.

An interesting example is an DPA article (mirrored by SZ.de) that reports about the then ongoing clearing of the village “Lützerath” to make way for the expansion of a lignite coal mine.^86^ The article has multiple citations from different activists, different politicians, and different representatives of law enforcement, which are all reported correctly by GPT-4. A single quotation by a press speaker of the mining company is missed however (i.e. not specified as “Business representative”). Moreover, the cited politicians are described as “ministers” but are not identified as government representatives. In an article by Bild.de,^87^ only activists are directly quoted, while politicians are referred to but not quoted; nevertheless, both activists and politicians are classified as being quoted. Finally, in a Taz.de article,^88^ activists, law enforcement, politicians, and government representatives are reported as being quoted. The article indeed contains direct and indirect citations of activists, of a state attorney’s office, and of politicians of governing and non-governing parties (of the state of Berlin). Compared to the DPA article, the governing body of Berlin (the “senate”) is explicitly mentioned multiple times, which may explain why the cited member of the senate is correctly identified with their roles as government representative.

#### A.4.16 Focus on motivation versus disruption

The exact wording of the prompt was:

In the filtered data, most articles were classified with a “focus mostly on disruption” (46.4%), followed by an “equal focus on disruption and motivation” (26.1%), “focus mostly on motivation” (16.7%), “only focus on motivation” (7.5%), and “focus only on disruption” (1.1%). Only for 2.2% of articles did GPT indicate that this question cannot be decided.

Instances of articles that receive the “focus only on motivation” rating are opinion pieces by climate activists^89^ or sympathetic opinion pieces by journalists.^90^ A DPA article mirrored by Welt.de^91^ that receives the classification “mostly focus on motivation” reports about an upcoming FFF climate strike and briefly cites activists. An article by SZ.de^92^ with a neutral rating cites FFF who criticise LG for polarising society. The “mostly focus on disruption” label is assigned to a Bild.de article^93^ that harshly criticises LG for their initial reaction to a lethal accident in which part of the first responders allegedly arrived late due to a street blockade by LG. The article also cites the activists briefly.

#### A.4.17 Focus across activist groups

The exact wording of the prompt was:

Given that very few articles were about climate action groups other than FFF and LG, if many of those that were about others also included FFF or LG, we focused on FFF and LG throughout this paper. We do not provide examples here since they would be obvious. For a detailed validation of this and all other questions, see Section A.6.

#### A.4.18 100 word summary

Unfortunately, we cannot publicly share the articles we analysed in this paper, since many of them are paywalled online. However, we requested a summary of at most 100 words for each article, which we can share. The exact wording of the prompt was:

### A.5 Filtering the news articles

The scraping described in Section A.2 led to a total of 9, 127 news articles for the climate activist groups FFF and LG in the years 2022 and 2023. For 71 articles, we could not locate the full text for a variety of reasons or we could not determine the date of publication of the article. We therefore excluded these articles. This left us with a total of $N = 9,056$ articles.

For the main analysis, we then subset these articles such that they only included articles that satisfy the following three criteria:

1. The article is a proper news article (Section A.4.1)
2. The article is mainly about climate activism (Section A.4.5)
3. The article is predominantly about either Fridays for Future or Last Generation (Section A.4.16)

We chose these filters because they ensure that each article is sufficiently relevant to the topic of climate activism in Germany. After filtering on these three conditions, we were left with 4, 216 news articles.

For SZ.de we were able to identify the articles that were mirrored from the Deutsche Presse Agentur (DPA) news agency. We also noticed that SZ.de published a very large proportion of such mirrored articles compared to other newspapers. We therefore decided to only include original articles of SZ.de in the main analysis. For more details on this decision and the results of our analysis for the DPA articles mirrored by SZ.de, see Section B.6.

After removing the DPA articles from SZ.de, we obtained our final sample for the main analysis consisting of 4, 216 news articles. Unless stated otherwise, results in the paper are computed on these news articles. Basic descriptive statistics of this final set of news articles are in Section B.1.

### A.6 Validation

We relied on GPT-4 to analyse the large amount of articles we have collected, since it would be infeasible to do so with human raters. Here, we present a validation showing that GPT-4’s judgements are comparable to human judgements. We do this in two steps. First, we focus on validating two questions we used to filter the articles, namely whether the article is (a) a proper news article and (b) mainly about climate activism. Second, we then validated all questions based on a sample from all articles that GPT-4 considered proper news article and mainly about climate activism. For the validation of the two filter questions, we randomly sampled 32 articles from the full set of articles. For the validation of all other questions, we randomly sampled 36 articles that GPT-4 classified as being a proper news article, mainly about climate activism, and either about LG or FFF. We then realised that some combinations of action group and newspaper were not filled, and so we sampled another 24 articles so that there were at least three articles for each action group and newspaper.

All authors independently assessed the articles in addition to GPT-4. We also formed an assessment by combining the human ratings via a majority vote. We focus on this comparison in the following. Note that we binarised the responses to the peaceful vs violent question, collapsing “Peaceful” with “Mostly peaceful” and “Somewhat violent” with “Violent”. We further aggregated the motivation vs disruption responses, collapsing “Focus mostly on motivation / disruption” with “Focus only on motivation / disruption”, which yielded a three-point variable with a neutral middle category (equal focus on motivation and disruption). We did this because we saw that GPT and human agreement was not high enough to support such a fine-grained distinctions. We used the collapsed outcomes in all analyses throughout the paper.

Table 1 shows that the agreement between GPT-4 and the majority vote is reasonably high. For the first validation step, we found that the majority vote agrees in 92% of cases with GPT-4 both that an article is a proper news article and in 89% of cases that it is mainly about climate activism (value outside parenthesis). In the second step of the validation, we found that the question with the lowest agreement is the one about the focus on motivation vs disruption. Note that for that question and the peaceful vs violent question, there was the option to respond that no judgement could be made based on the article (for example because it simply did not provide sufficient information for such a judgement; see Section A.4). The values in the parenthesis for these questions indicate agreement between GPT-4 and human raters as to whether a judgement can be made at all, while the values outside the parenthesis indicate the agreement for the articles for which both GPT-4 and the human raters agreed a judgement can be made. We find that while the majority vote agrees with GPT-4 in 79% of the cases when the motivation vs disruption question could be decided, in only 60% of the articles GPT-4 and the majority vote agrees that the question can in fact be decided.

Table 1 also shows baselines in the response of GPT-4 to the 60 articles of the second validation. For example, we find that 18% of articles are mainly about climate change, which makes sense given that these articles should mainly be about climate activism (which 100% of them are), and that most articles are reports (72%) and about protests (48%). These percentages are computed across newspapers and climate activist groups, and they reflect the results provided in the main text of this paper, except for the relative frequency of Fridays for Future and Last Generation, since we aimed to balance the number of articles that are about the two respective groups.

**Table 1:** Agreement between raters on all question in the first and second validation

Question	AI-SW	AI-FD	AI-JH	AI-Majority	H-A	AI Baseline
Proper news article	0.79	1.00	0.83	0.92	0.99	1.00
	(1.00)	(0.98)	(0.98)	(0.98)		
Article type	0.88	0.86	0.88	0.89	0.90	Report: 0.72, Interview: 0.07, Opinion piece: 0.20, Other: 0.02
Article about	0.79	0.79	0.79	0.77	0.84	Protest: 0.48, Legal issues: 0.13, Other: 0.38
Broadly activism	0.96	1.00	0.96	0.98	0.96	1.00
Mainly activism	0.88	0.88	0.96	0.89	0.92	1.00
	(0.86)	(0.91)	(0.84)	(0.91)	(0.90)	
Broadly climate	0.91	0.84	0.75	0.88	0.77	0.82
Mainly climate	0.80	0.80	0.80	0.79	0.92	0.18
Portrayed as threat	0.77	0.75	0.64	0.70	0.86	0.53
Risks mentioned	0.77	0.75	0.66	0.71	0.83	0.33
Solutions mentioned	0.86	0.73	0.82	0.89	0.73	0.38
Disruption mentioned	0.88	0.80	0.79	0.88	0.80	0.80
Portrayed as violent	0.82	0.85	0.79	0.86	0.79	Peaceful: 0.53, Violent: 0.32, Not applicable: 0.15
	(0.80)	(0.79)	(0.77)	(0.86)	0.79	
Portrayed as helpful	0.79	0.84	0.82	0.82	0.77	Helpful: 0.04, Unhelpful: 0.12, Not applicable: 0.85
Portrayed as polarising	0.71	0.82	0.73	0.77	0.79	0.28
Actors quoted	0.85	0.83	0.86	0.86	0.87	Activist: 0.54, Politician: 0.39, Government repr.: 0.23, Law enforcement: 0.20, Business repr.: 0.11, Expert or scientist: 0.09, Lawyer of activists: 0.02
Motivation vs disruption	0.75	0.77	0.62	0.79	0.71	Motivation: 0.28, Equal Focus: 35, Disruption: 0.35, Not applicable: 0.02
	(0.61)	(0.69)	(0.62)	(0.60)	(0.71)	
Climate group	0.86	0.84	0.77	0.84	0.84	FFF: 0.42, LG: 0.58

Numbers in parenthesis for ‘proper news article’ and ‘mainly about climate activism’ refer to the second validation. Numbers in parenthesis for ‘peaceful vs violent’ and ‘motivation vs disruption’ give the agreement about whether both raters said the article has enough information to provide a judgement of not; numbers outside the parenthesis give the agreement when both raters in fact gave a rating. For the actors quoted, we computed agreement for each actor separately and then averaged agreement over actors. The column ‘H-A’ gives the average pair-wise agreement between all human raters. For items with values in and outside parentheses, AI baselines refer to the validation with 60 articles (compared to the first validation of two filter questions, which used 32 articles)

### A.7 Limitations of GPT-based analysis

Employing GPT-4 allowed us to accurately extract key information about the portrayal of climate change and climate activists from a large set of articles about climate activists in Germany. However, in the development phase of this project it became clear that GPT-4 only showed good performance in extracting relatively literal and matter-of-fact information. This means that a large amount of nuance about how different journalists and newspapers are portraying different climate activist groups remains unexplored by our large-scale quantitative analysis.

Journalists have many nuanced tools at their disposal that allow them to clearly position themselves for or against a certain group or issue without being literal about it. These tools include word choice, representing only one perspective by laying out only one argument or only citing individuals representing one camp, selectively presenting data or quoting individuals who do so, choosing a negative framing, and many more. Here we give just one example of an article to showcase all the nuance that is missed by our GPT-4-based analysis. We focus on the article “Fridays for Future haben die Letzte Generation gezüchtet” (English: “Fridays for Future bred Letzte Generation”) by Nikolaus Blome for Spiegel.de.^94^ There are at least the following ways in which Nikolaus Blome portrays climate activist in a negative light, and which are not captured by our GPT-4-based analysis:

1. He states that FFF engages in “Maximalismus” (English: “maximalism”), which in German means that they do not tend to accept compromises, which might be seen as unreasonable by a large section of the general public.
2. The author writes “In ihrem Ruecken schlendern die überwiegend jungen Demonstranten im Sonnenschein vorbei, [...]”. The verb “schlendern” (English: stroll, linger) suggests that the activists are engaging in a leisurely and potentially non-serious activity.
3. The author writes about Luisa Neubauer, a leader of FFF: “Sie wirkt dabei wie erleichtert, doch es ist Selbsthypnose: [...]” (English: “She seems relieved, but it’s self-hypnosis: [...]”). Suggesting that Luisa Neubauer is engaging in self-hypnosis seems to undermine her credibility and professionalism.
4. The author writes “Diese Suche sollte vor die eigene Haustür führen, finde ich, doch das wird sie garantiert nicht.” (English: “I think this search should lead to your own front door, but it definitely will not.”). The author implies that FFF made mistakes, and that they are unable to candidly reflect on possible mistakes and accept those. The author thereby seems to portray the activists as immature.
5. The author goes on to describe the climate activists of LG as “[...] die zukunftszerquälten, schmerzbefreiten Aktivisten der Letzten Generation sein.” (English: “[...] be the future-tortured, pain-free activists of Last Generation.”), which can be read as sarcastic and mocking remark about the climate activists of LG.
6. The author writes “Im Folgenden wollen sie in der Hauptstadt endlos Rabatz machen, bis die Regierung Umkehr gelobt, was immer das ist.” (English: “Afterwards, they want to rage endlessly in the capital until the government vows to turn around, whatever that is.”) By choosing the words “Rabatz machen” (English: “raging”) and saying “whatever that is” the author deligitimizes the activists and suggests that they do not have clear goals.
7. The author writes “Konstant um die 80 Prozent der Bevoelkerung geht das Selbstgeklebe auf die Nerven.” (English: “Around 80 percent of the population finds self-adhesive annoying.”). The author here misrepresents both the numbers and the wording of a Civey survey in 2022,^95^ making the statement much stronger than is warranted by the actual source.
8. The author writes “Frau Neubauer hat eitel uebertrieben [...]” (English: Ms. Neubauer vainly exaggerated [...]) and thereby implies that Luisa Neubauer, a leader of FFF, is vain.
9. The author writes “Frau Neubauer hat eitel übertrieben, als sie in dem Interview sagte, dass man ‘hart gekaempft’ habe, dass es nun ‘einen Konsens für den Klimaschutz gibt’. Und in einem weiteren, dass die Fridays ‘die Klimakrise von einem Nischenproblem zu einem Gesellschaftsproblem gemacht’ habe. Wirklich, das waren alles die jungen Leute allein?” (English: “Ms. Neubauer vainly exaggerated when she said in the interview that they had ‘fought hard’ so that there was now ‘a consensus for climate protection.’ And in another, that the Fridays had ‘turned the climate crisis from a niche problem into a social problem.’ Really, was it all the young people alone?”) and thereby misrepresents Luisa Neubauer, who only says that FFF were “fighting hard” for “a consensus for climate protection”. The author misrepresents her by saying that she claimed that FFF was the only cause for that, which is not what Luisa Neubauer said.
10. The author writes “Sie waren es, die die Verabsolutierung symbol-pittoresker Kleinigkeiten erfanden: ‘Hambi’, ‘Lützi’ und die anderen Lieblinge aus dem Streichelzoo der Bewegung.” (English: They were the ones who invented the absolutization of symbolic, picturesque little things: ‘Hambi’, ‘Lützi’ and the other favorites from the movement’s petting zoo.) Here the author implies that the confrontations the climate activists of FFF engaged in were “little things”. The expression “[...] the movement’s petting zoo” could also be seen as belittling the climate activists.
11. Niklaus Blome also writes “Ebenfalls geht die Verächtlichmachung des Staates und ‘der Politik’ auf die Fridays zurück, [...]” (English: “The despising of the state and ‘politics’ also goes back to the Fridays, [...]” and thereby suggests directly that FFF despises the state, which is factually mistaken.

While we were not able to capture such nuance with the methodology employed in this paper, using human readers or/and a more elaborate GPT-4-based analysis would provide a more complete picture of the portrayal of climate change and climate activist groups in the news media. We consider this type of in-depth analysis a promising area for future research, especially as artificial intelligence advances even further.

### A.8 Scraping press releases for actions

To identify the dates of the groups’ actions, we analysed official press releases of both groups. At the time of writing, both groups provided a full history of these on their websites.^96^ Given that the number of press releases from 2022 and 2023 was much lower for FFF (around 50 versus almost 400 for LG), we could easily identify the main actions by reading the press releases one-by-one. Note that we only cover nation-wide protests, not smaller decentralised actions for FFF. For LG, we extracted the individual press releases from the HTML source code. Similarly to our main GPT-based analysis (Section A.4), we used prompts to identify whether a press release was primarily concerned with an action, as well as to extract some meta-information. This is the text of the full prompt we used:

### A.9 Estimating electricity consumption of the GPT-4 analysis

Unfortunately, OpenAI does not provide data on electricity consumption for running their models. Therefore, we have to resort to a best-effort estimation. A clear estimate of the energy used per processed token has not been given for GPT-4 in the literature either (Faiz et al. 2024). A simple estimate that has been used for GPT-3 stems from a statement of OpenAI’s Sam Altman that suggests a worst-case cost estimate of $0.09 per query. Assuming that 50% of this is used for energy, an energy price of $0.15 per kWh, and 1000 tokens to be processed per request, one arrives at 0.3 Wh per token^97^. Since this quantity is not available for GPT-4 Turbo, we use the ratio of cost per token between the two models as a proxy for the ratio of energy used per token. Table 2 shows the calculation of our estimate for the energy used to compute the results reported in this paper (accounting only for inference, not training). We arrive at an estimate of 27 kWh, which is about the same as using a modern refrigerator for 66 days.

**Table 2:** Energy estimation for GPT-4 Turbo inference

Description	Value
Average number of tokens per article	860.0
Number of articles	4216
Total tokens processed	3, 625, 760
Energy per token	0.0003 kWh
Total energy consumed (GPT-3)	1, 088 kWh
GPT-4 Turbo pricing factor	0.025
Total energy consumed (GPT-4 Turbo)	27.2 kWh

## B Sectional analyses

### B.1 Extended descriptive statistics

Table 3 shows the number of articles published in the seven newspapers for the two climate activist groups after filtering. We see that there are overall more articles about LG than about FFF. However, we also see that the proportion differs across newspapers. For example, while Bild.de published 8.44 times more articles about LG than about FFF, Spiegel.de published only 1.75 times more articles about LG.

**Table 3:** Number of published articles in the seven newspapers about the two climate activist groups

	Taz.de	Zeit.de	SZ.de	Spiegel.de	FAZ.net	Welt.de	Bild.de	$\Sigma$
Fridays for Future	26	26	36	58	113	124	91	474
Last Generation	408	136	213	227	322	662	768	2736

Table 4 shows the number of different types of articles in the seven newspapers. For all newspapers, the large majority of articles are news reports. However, there is considerable variation across newspapers in terms of types of articles. For example, while 23% of articles in Taz.de are opinion pieces, only 5% and 6% of articles of Bild.de and Welt.de are opinion pieces, respectively.

**Table 4:** Number of different types of articles published across the seven online newspapers

	Taz.de	Zeit.de	SZ.de	Spiegel.de	FAZ.net	Welt.de	Bild.de
Report	248	809	236	202	103	710	398
Interview	33	16	7	22	12	18	1
Opinion Piece	100	31	37	24	45	41	25
Other	54	2	5	1	2	17	10

Table 5 shows the number of different types of articles published about the two climate activist groups. While for both climate activist groups most articles are reports, there are differences between the proportion of different article types across groups. For example, 8% of articles about FFF are opinion pieces, while only 3% of articles about LG are opinion pieces.

**Table 5:** Number of different types of articles published across two climate activist groups

	Report	Interview	Opinion Piece	Other	$\Sigma$
Fridays for Future	367	41	39	27	474
Last Generation	2339	262	70	64	2735

Table 6 shows how the topic of reporting differs for the two climate activist groups. We see that the reporting about LG is dominated by reports about protests, while articles about FFF are more often in the category “Other”, which includes interviews and opinion pieces. There is also a large number of articles discussing legal issues connected to LG, while there are few articles of such type for FFF.

**Table 6:** Number of articles published across different topic categories and climate activist groups

	Fridays for Future	Last Generation
Reporting about a protest	278	1753
Reporting about legal issues	13	467
Other	183	516

### B.2 Article length across newspapers

Figure 4 displays violin plots of the distributions of article lengths (in number of words) across the seven newspapers considered in the main text. We see that Zeit.de, Bild.de, and Welt.de articles tend to be shorter than articles in the other four newspapers. We also see that articles in Zeit.de, Bild.de, and Welt.de tend to be relatively similar in their length, while there is more spread in the remaining newspapers. In particular, the remaining newspapers publish a small but consistent amount of longer articles, as can be seen in the longer tails for those newspapers.

### B.3 Variation across activist groups and newspapers

Here we assess the variation across climate activist groups and newspapers. To compute the former, we took the values in Fig. 2 and, for each outcome, computed the pairwise differences between FFF and LG for all newspapers. This yielded seven values, for which we computed the standard deviation, which is visualised in the left panel of Fig. 5 in red. To compute variation across newspapers, we computed, for each outcome and climate activist group, the standard deviation of the values in Fig. 2. We averaged the standard deviation across climate activist groups, which yielded the blue bars in the left panel of Fig. 5. We repeated this exercise for the values corresponding to the portrayal of climate activists. The result is shown in the right panel of Fig. 5.

Looking at the portrayal of climate change, we found that the variation across newspapers was considerably larger for “portrayed as a threat” and ‘solutions mentioned’, with much smaller differences for “broadly about climate change” and climate crisis terms. The variation for “risks mentioned” was larger between activist groups than between newspapers. Looking at the portrayal of climate activist groups, we found that the variation across newspapers was larger for negative terms, and the questions of motivation vs. disruption and being portrayed as peaceful vs. violent. The variation across activist groups, on the other hand, was larger for whether disruption was mentioned and whether activists were portrayed as polarising.

### B.4 Correlation of outcomes

We computed Kendall’s $\tau$ between all outcomes across newspapers and climate activist groups to assess their similarity (Fig. 6). A number of correlations stand out. First, an article being broadly about climate change is correlated with it portraying climate change as a threat; mentioning it risks and solutions; mentioning climate crisis terms; and focusing more on the motivation of activists rather than the disruption they cause. Similarly, an article is more likely to be broadly about climate change when it does not mention any disruption and when activists are being portrayed as peaceful and without negative terms. The strongest correlation of whether climate change is being portrayed as a threat was with the climate crisis terms, which is due to the fact that we affirm the former if terms like “climate catastrophe” or “climate crisis” are in the article (see also Section A.4.8). In terms of agreement, the answers to the threat and climate crisis terms questions are the same in 87.1% of articles. If climate change is portrayed as a threat, then there is a 69.7% chance that crisis terms are also mentioned, and 93.2% for the other way around.

The threat questions is also correlated with whether any risks of climate change are mentioned, but less than one might think. In terms of actual agreement, in 79% of articles the threat and risk questions provide the same answer, but if climate change is portrayed as a threat then there is only a 44.5% chance that risks are also mentioned. The answer to the risk question is in turn correlated with whether solutions are mentioned, which suggests that articles that provide information on climate change do so along multiple dimensions. Whether a disruption is mentioned is negatively correlated with whether the article focuses more on the disruption caused or the motivation of the activist, which makes perfect sense; in the main text, we look at the latter question only for articles where any disruption has indeed occurred.

If activists are portrayed as more violent, less information about climate change is provided along multiple fronts (threat, risks, solutions). The polarisation question we put to GPT-4 (see also Section A.4.14) is correlated with the frequency analysis to some extent, but the actual agreement between them is considerably higher at 84.2%. There are many cases (625 articles, or 14.9%) where GPT-4 assesses the article as portraying climate activists as polarising while none of the polarisation terms we came up with are mentioned; the converse is much less often the case (35 articles, or 0.83%). This suggests GPT-4 is more liberal in its answers (and thus likely catching more true positives) than our frequency analysis.

### B.5 Actors quoted in articles

The content and framing of a news report naturally highly depends on the content and framing on the sources it draws from. Therefore, the question has been raised to what extent newspaper reports are influenced by readily available sources (e.g., statements by government, police, or business representatives) versus sources that are harder to obtain (e.g., statements by experts or scientists; Bennett 1990; Livingston and Bennett 2003; Tiffen et al. 2014). To contribute to this discussion, we extracted for each news article which actors are being quoted. Figure 3 shows the proportion of articles that include different actors for the different newspapers, separately for FFF (left panel) and LG (right panel). Overall, we see that activists and politicians are the most frequently quoted actors in articles on climate activism. Interestingly, experts or scientists are very rarely quoted despite the fact that they would arguably have most expertise to discuss facts around the topic the activism is about.

Comparing the two activist groups, we see that the activists of LG are much less often quoted than the ones of FFF. At the same time, politicians and especially representatives of law enforcement are quoted a lot more. All newspapers except Taz.de and FAZ.net quote representatives of law enforcement much more frequently when reporting on LG compared with FFF. This increase is especially marked for Bild.de and Welt.de.

### B.6 Comparing original SZ.de With DPA-Mirrored articles

For SZ.de we were able to obtain the information whether an article was written by SZ.de journalists themselves or whether it has been copied from the news agency Deutsche Presse Agentur (DPA), which is a major global press agency and the biggest press agency in Germany. We also noticed that SZ.de copies these article much more than any of the other newspapers. We therefore chose to exclude these DPA articles from the main analysis to avoid the possibility that they obscure differences across newspapers.

Here we repeat the main analyses in the paper but distinguish between between articles on SZ.de which have been written directly by its journalists and articles that are acquired from DPA. Figure 9 shows that DPA articles about climate activist groups less frequently include climate crisis terms, portray climate change less frequently as a threat, and mention risks and solutions less frequently. The differences are more pronounced for reporting on LG than for reporting on FFF. Overall, this may be due to the fact that DPA is typically reporting about specific events, while most other online newspapers also include opinion articles and interviews.

Figure 9 shows the portrayal of climate activist groups themselves. Here, we observe no differences between native SZ.de and DPA articles in whether disruptions of activist groups are mentioned. However, DPA articles focus more on disruption, portray activists as more violent but less polarising, and use negative terms less often (when reporting on FFF).

Figure 10 shows the actors being quoted in news articles. For FFF, DPA tended to quote activists, government representatives, and law enforcement more and politicians, business representatives, experts or scientists, and lawyers of activists less frequently. For LG, DPA tended to quote activists, business representatives, experts or scientists, and lawyers of activists less and law enforcement and government representative more frequently than native SZ.de articles. Politicians were quoted an equal amount of times.

### B.7 Main results only for articles reporting on protest

We have shown in Section B.1 that the proportion of articles that report about a protest is higher amongst articles about LG compared to articles about FFF. Here we repeat our main analysis, but using only those articles that are reporting about a protest. This is interesting, because it allows us to determine to what extent differences in reporting about groups is due to the fact that articles about them tend to be about different topics.

We find that the differences between the two activist groups generally tend to be smaller when only focusing on articles that focus on a protest. However, differences remain, which point to that the type of action the article reports about (e.g., climate strike for FFF versus blocking a road for LG) and possibly additional factors influence the way newspapers report on climate activist groups and climate change.

### B.8 Main results split for years 2022 and 2023

Here we investigate to what extent the main results about the portrayal of climate change and climate change activists was different in the years of 2022 and 2023.

Figures 14 and 15 show the same results as Fig. 2 in the main text, but subset articles from only 2022 and 2023, respectively. We see the overall patterns both in terms of the proportions across questions, and the results across activist groups and newspapers are similar in both years. The largest changes are in the portrayal of climate change in articles about Fridays for Future, where all newspapers except FAZ.net mention risks and solutions less in 2023 than in 2022.

Figures 16 and 17 show the same results as Fig. 3 in the main text, but subset articles from only 2022 and 2023, respectively. Again, we see that the results in terms of the proportions across questions and in the differences between groups and newspapers are similar in 2022 and 2023. The largest difference is again in the articles about Fridays for Future: most newspapers, and especially right-leaning newspapers mention disruption more often in 2023 and also put more focus on disruptions relative to the motivation of the protest in 2023.

## Footnotes

1 https://www.dwdl.de/zahlenzentrale/95133/bild_und_welt_fuehren_konvergenzranking_der_zeitungen_an/

2 https://gruppe.spiegel.de/news/pressemitteilungen/detail/der-spiegel-ist-reichweitenstaerkste-kaufzeitschrift

## Materials availability

The code to reproduce all results is available at https://github.com/jmbh/ClimateActivistCoverage.
